# Human perception of art in the age of artificial intelligence

**DOI:** 10.3389/fpsyg.2024.1497469

**Published:** 2025-01-08

**Authors:** Jules van Hees, Tijl Grootswagers, Genevieve L. Quek, Manuel Varlet

**Affiliations:** ^1^The MARCS Institute for Brain, Behaviour, and Development, Western Sydney University, Penrith, NSW, Australia; ^2^School of Computer, Data and Mathematical Sciences, Western Sydney University, Penrith, NSW, Australia; ^3^School of Psychology, Western Sydney University, Penrith, NSW, Australia

**Keywords:** visual perception, generative AI, DALL·E, artworks, appreciation, discrimination

## Abstract

Recent advancement in Artificial Intelligence (AI) has rendered image-synthesis models capable of producing complex artworks that appear nearly indistinguishable from human-made works. Here we present a quantitative assessment of human perception and preference for art generated by OpenAI’s DALL·E 2, a leading AI tool for art creation. Participants were presented with pairs of artworks, one human-made and one AI-generated, in either a preference-choice task or an origin-discrimination task. Results revealed a significant preference for AI-generated artworks. At the same time, a separate group of participants were above-chance at detecting which artwork within the pair was generated by AI, indicating a perceptible distinction between human and artificial creative works. These results raise questions about how a shift in art preference to favour synthetic creations might impact the way we think about art and its value to human society, prompting reflections on authorship, authenticity, and human creativity in the era of generative AI.

## 1 Introduction

Artificial Intelligence (AI) has become ubiquitous in our everyday lives. With each iteration in technological capabilities, the gap between AI and human ability seems to narrow. One such advancement has been the recent wave of image-synthesis models; AI image-generation tools that have evolved to a level of sophistication such that it is nearly impossible to distinguish between photographs of real human faces and those generated by a computer (Moshel et al., 2022; Nightingale and Farid, 2022). The fact that AI is able to fool the human visual system’s perception of faces—one of our most deeply-rooted and evolutionarily relevant brain functions—is certainly cause for concern (Westerlund, 2019), but how does it fare against what has arguably been the cultural benchmark of human creativity throughout history: Art?

Addressing this question has increasing importance for understanding the changing landscape of the art world and the role of technology in shaping artistic production and consumption. Separately from concerns that centre on questions of intellectual property and privacy violations (Smits and Borghuis, 2022; Zhou and Nabus, 2023), AI-generated art also raises fundamental questions about how we might (re)define creativity (Epstein et al., 2023; Oksanen et al., 2023), an ability considered until now to be essentially human-specific. This issue has captured interdisciplinary interest, with researchers across computer science, philosophy, and psychological and social sciences joining forces to investigate how we process and interact with the AI-generated works that we are increasingly exposed to in advertising, social media, and scams (Baidoo-Anu and Ansah, 2023; Epstein et al., 2023; Millet et al., 2023; Oksanen et al., 2023; Ooi et al., 2023).

Previous research has shown a consistent trend of negative bias towards AI-generated art (Agudo et al., 2022; Chamberlain et al., 2018; Chiarella et al., 2022; Gangadharbatla, 2022; Horton et al., 2023; Millet et al., 2023; Ragot et al., 2020), however, this research has primarily examined the impact of knowing whether the art was created by AI or humans (rather than assessing artworks’ intrinsic aesthetic qualities). Whether or not humans exhibit a reliable preference for the *intrinsic aesthetic qualities* of (unlabelled) human- or AI-generated art has received comparatively less attention. Therefore, there is lack of understanding in current literature of how people respond to AI art separately from biases related to authorship attribution, which will be increasingly important as AI-generated art continues to proliferate, and contextual information such as authorship attribution becomes more scarce.

Here, we aimed to address this critical gap in the literature by examining observer preferences for human-made *vs.* AI-generated art in the absence of any knowledge of the artworks’ origins. The novelty of this study also resides in providing an objective and quantitative assessment of the human perception of artificial art made using OpenAI’s DALL·E 2, one of the most advanced AI tools for art generation.^1^ With high-level performance for representational image generation, DALL·E 2 represents a step change in the field, as technology and synthetic representational artworks publicly accessible before its release were often much more rudimentary in complexity of composition and general verisimilitude (Pan et al., 2019). Here we focus on how observers perceive and appreciate the aesthetic qualities of DALL·E 2 artworks by comparing human observers’ appreciation of unlabelled AI-generated and human-made art and testing observers’ ability to distinguish between the two.

**Figure 1:**
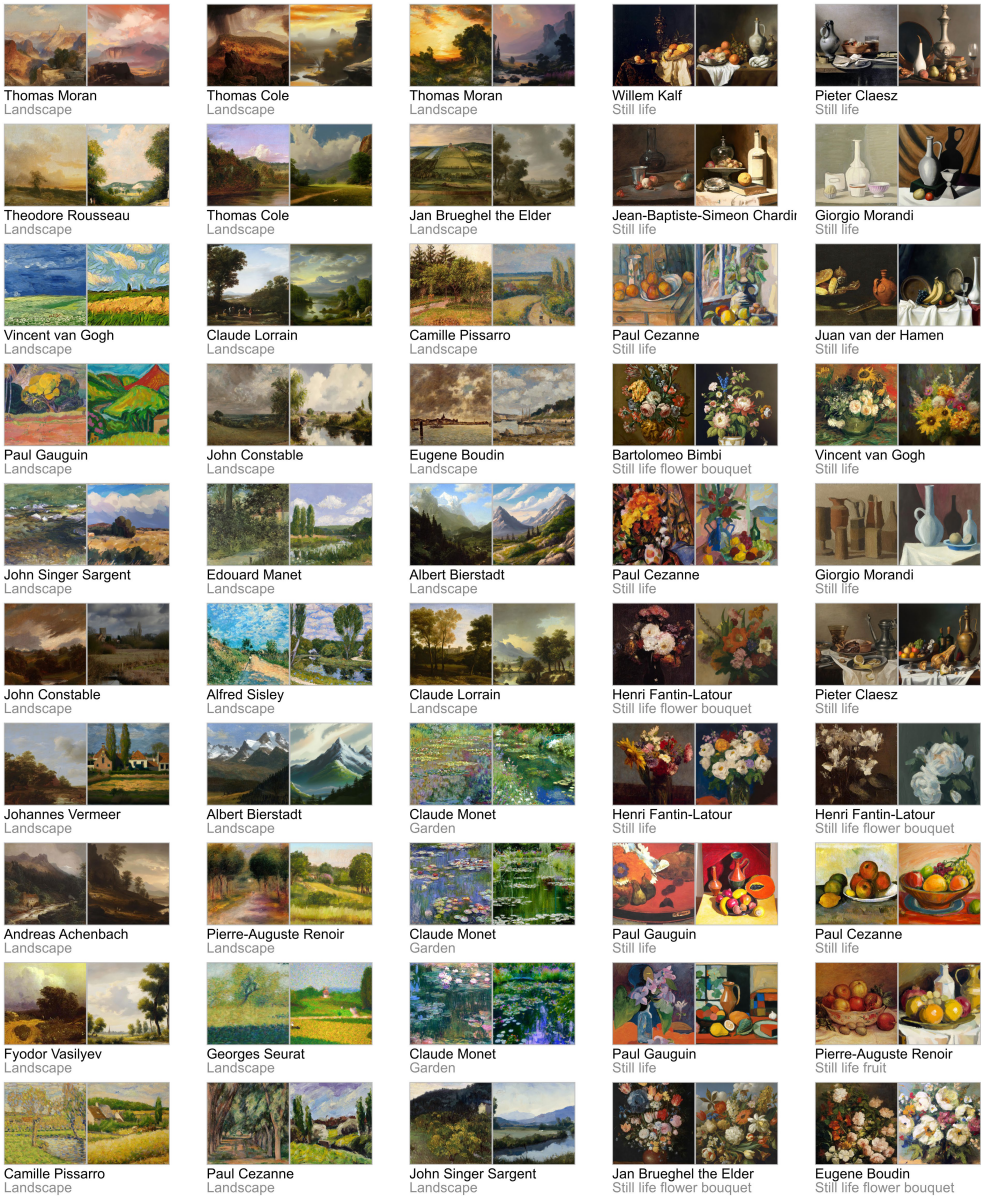
The 50 pairs of human-made (left image) and AI-generated (right image) artworks used in Experiments 1 and 2. Corresponding author and style used as prompts in DALL·E 2 appear below each pair. Images of human-made artworks were sourced from Wikimedia Commons (https://commons.wikimedia.org) and WikiArt (https://www.wikiart.org); AI-generated images were obtained from DALL·E 2 (https://openai.com/dall-e-2).

**Figure 2:**
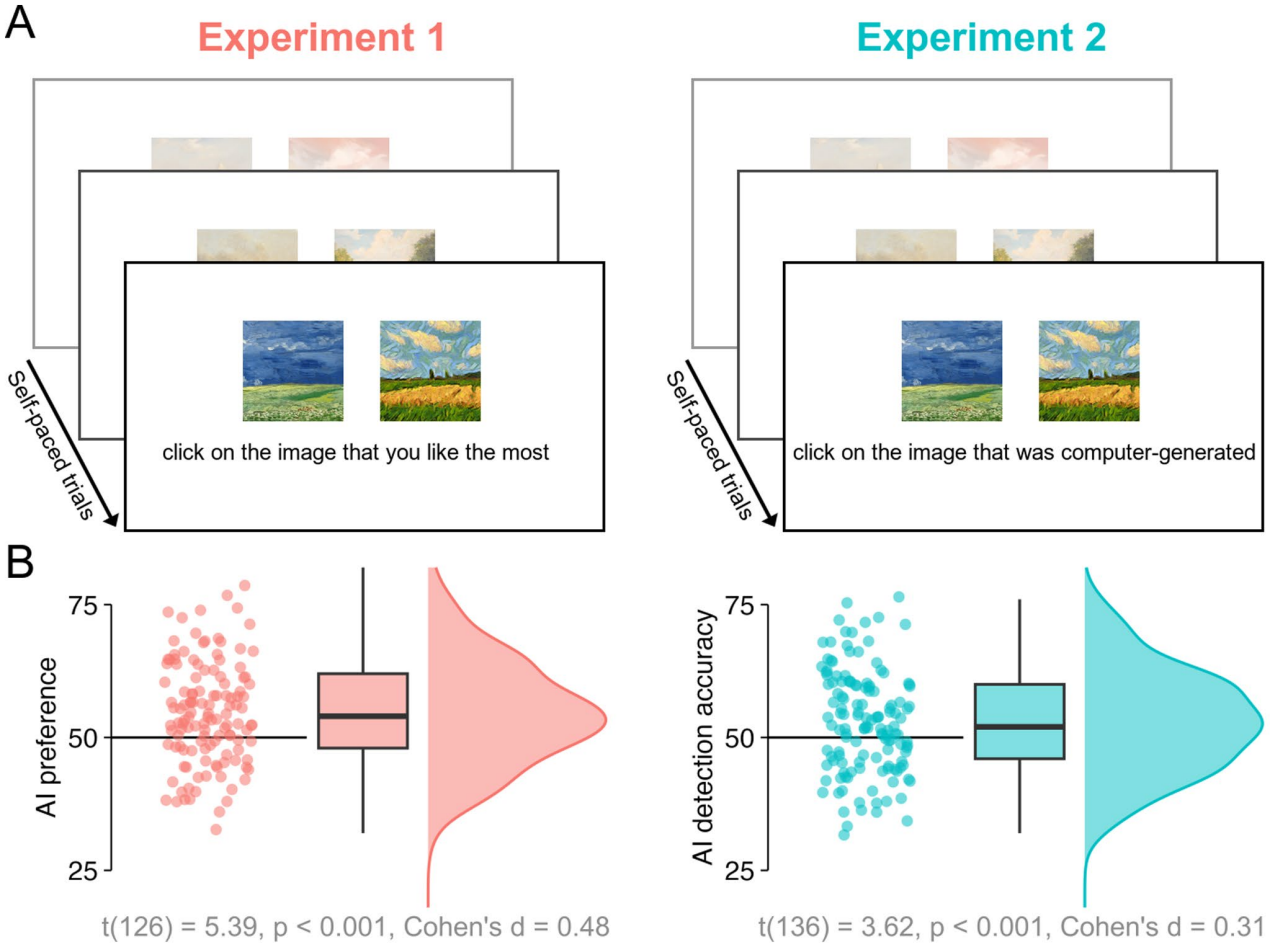
**(A)** Illustration of task displays used in Experiments 1 and 2 to, respectively, examine AI preference and AI-detection accuracy. There were 100 trials in each experiment, 50 of which were the critical human-AI pairs **(B)** Individual participants’ AI preference scores (Experiment 1, at left) and AI detection accuracy scores (Experiment 2, at right) averaged across all pairs of artworks. Corresponding boxplots and distributions appear at right.

To assess human observers’ appreciation and discrimination of non-abstract artworks generated by both humans and AI, we paired 50 lesser-known real artworks by famous representational artists (absent in the WikiArt’s “famous work” section) with 50 artificial artworks generated in a similar style using OpenAI’s DALL·E 2 with comparable visual features (e.g., colour, style, composition), as depicted in Figure 1. Online observers viewed these image pairs in either a preference judgement task (Experiment 1, *“Which artwork do you like the most?”*, 127 participants) or a real-artificial discrimination task (Experiment 2, “*Which artwork was generated by a computer?”*, 137 participants) (see Figure 2A). To minimise bias and conceal the true purpose of each experiment (i.e., comparing human- vs. AI-generated), in both cases the 50 matched image pairs appeared randomly intermingled with random pairs (e.g., Human-made vs. Human-made) drawn from the full image set. We also assessed online observers’ art experience to test its potential influence on the appreciation and discrimination of these images.

## 2 Methods

### 2.1 Participants

Online participants from Western Sydney University were recruited via the university (SONA) participant management system in exchange for course credits. We recruited 127 participants in Experiment 1, including 31 males, 95 females, and 1 non-binary with mean age of 22.27 (SD = 5.89), and 137 participants in Experiment 2, including 26 males, 109 females, and 2 non-binary with a mean age of 21.76 (SD = 6.99). Our participants reported on average a medium level of expertise in art, with subjective ratings of interest in art of 63.32 (SD = 29.68) and 62.22 (SD = 28.12), knowledge of art history of 30.81 (SD = 25.41) and 28.41 (SD = 26.46), artistic personality of 53.32 (SD = 28.35) and 52.49 (SD = 29.00) on a scale of 0 to 100, in Experiment 1 and 2, respectively.

The study was approved by the Human Research Ethics Committee of Western Sydney University. All participants provided written informed consent prior to the study. The experiment was performed in accordance with the Declaration of Helsinki and relevant guidelines and regulations for research involving human research participants.

### 2.2 Stimuli

Stimuli in Experiments 1 and 2 were 50 images of real artworks and 50 images of AI-generated artworks representative of various artistic styles (Baroque, Romanticism, Impressionism and Post-impressionism). Each image was presented at 200 × 200 pixels, which, assuming a standard laptop screen, corresponds to approximately 6 × 6 degrees visual angle (note this varies depending on the participant’s own device). Real and synthetic artworks were matched across artistic styles to form 50 pairs of images shown in Figure 1. The real images were 50 lesser-known artworks by famous representational artists retrieved from Wikimedia Commons^2^ and WikiArt.^3^ The selection process involved manually skimming through the publicly accessible catalogues of artists whose works have proliferated to the point that they are significantly represented in the training data of DALL-E 2. Since there is no direct metric to measure the degree of popularity for many a given work, selection was made based on the following criteria: Absence in the “famous work” section on WikiArt and whether the work could be reasonably assumed to be representative of the associated artist’s style (e.g., While Vincent van Gogh’s catalogue features many charcoal drawings, these were excluded as they do not fit with the general perception of his art style).

The AI-generated stimuli were created with DALL·E 2, an image diffusion model that generates high-quality, complex images based on textual prompts input by the user.^4^ Briefly described, this process relies on a text encoding model (Contrastive Language-Image Pre-training; CLIP) to link textual input to visual output by use of a two-stage model involving a “prior” image caption embedder and an “encoder,” which work in tandem to extract information relevant to the desired visual output (Ramesh et al., 2022). After sufficient training, the CLIP model is frozen and the now-embedded semantic information it produced is used to train a diffusion ‘decoder’ that allows for the process to be inverted. DALL·E 2 employs a diffusion model named Guided Language to Image Diffusion for Generation and Editing, which after training, allows for text-conditional image generation. This is achieved by training a Markov chain to make certain inferences using a set of sample images, which are iteratively provided with more Gaussian noise until it is able to reverse the generation process (Ho et al., 2020). This model is then trained using a generative adversarial network (GAN), where two networks, a generator and a discriminator, are locked in a zero-sum game and continually pushed to greater levels of image generation refinement (Poltronieri and Hänska, 2019). The result is a highly accessible and versatile AI image-generation tool that can convert textual prompts into detailed realistic images.

DALL·E 2 was used with 36 unique prompts that included both an artist’s name and the type of artwork (e.g., “Paul Cezanne style still life painting,” see Figure 1). DALL-E generates several images in response to each prompt. Several prompts were used multiple times (e.g., Claude Monet style garden painting). To minimise bias in the selection process, the generated images were manually compared to famous artists’ works found on Wikimedia Commons^5^ and WikiArt,^6^ focusing on comparable visual features (e.g., colour, style, composition) before inclusion. As with the real images, the primary selection criterion was whether a given outputted image could reasonably be assumed to be representative of the respective artist’s style. Manual selection was employed to remove clear outliers, and the selected images were then cropped to remove the DALL·E logo in the bottom-right corner.

To account for selection bias, image statistics were analysed using a GIST image descriptor in MATLAB, focusing on overlapping low-level visual similarities within each category (i.e., AI- or human-made stimuli). This could influence overall appraisal, e.g., should the selected AI stimuli possess a particular visual feature that the human stimuli do not have, this may result in one category being judged as more or less visually appealing as a whole to a given participant. The GIST descriptor greatly reduces image size and quality to rudimental features to compare input data for overlapping features (Oliva and Torralba, 2001). The results of this statistical image analysis were then mapped onto a matrix, which showed only a minor overlap between categories and was thus deemed too small to induce potential biases. We also calculated aposteriori luminance, root mean square contrast and entropy to test their potential contribution to our results (individual image values and category distributions are presented in Supplementary material).

The experiment ran online in participants’ web browsers (Grootswagers, 2020), was coded using the javascript framework jsPsych, version 7.3 (De Leeuw, 2015), and ran on Pavlovia (Peirce et al., 2019).

### 2.3 Procedure

Experiments 1 and 2 had the same experimental procedure and design, differing only in terms of participant instruction. At the start of the experiment, participants reported their demographic information, along with their art expertise, operationalised through three questions: (1) “*Rate your interest in art*,” (2), “*Rate your knowledge of art history*,” and (3) “*How artistic are you?*” Participants indicated their response using a slider coded to a value between 0 and 100. Next, in the main part of the experiment, the 50 pairs of artworks shown in Figure 1 were presented once in a random order with the human-made and AI-generated stimuli being randomly presented either on the left or right side. Participants in Experiment 1 were not aware of the true aim of the study. They were not informed of the origin of the artworks and were simply instructed to select which one of the two images they preferred (see Figure 2A). Participants in Experiment 2 were told that one in each pair was AI-generated and instructed to click on it (see Figure 2A). Fifty additional trials with unique 50 pairs randomly drawn from the 100 (human and AI) artworks were included in each experiment (but not analysed) to ensure that participants remained naïve to the experimental manipulation. The 100 trials in total were performed by participants at a self-selected pace. Each pair of images remained onscreen until a selection was made. The total duration of the experiment was about 5 min. Participants could only participate in either Experiment 1 or Experiment 2 to ensure participants in Experiment 1 were not aware of the presence of AI-generated stimuli.

### 2.4 Data and statistical analysis

AI preference scores from Experiment 1 and AI detection accuracy scores from Experiment 2 of each participant were averaged across the 50 pairs of stimuli and then submitted to one-sample *t*-tests to examine deviations from the 50% chance level. The scores for each pair of images were also averaged across all participants within each experiment separately to test the image-wise correlation between the two experiments using Pearson correlations. A principal component analysis was conducted on the three expertise scores and data on the first dimension (71 and 76% variance explained in Experiment 1 and 2, respectively) were used to test the effect of expertise on AI preference and AI detection accuracy in Experiment 1 and 2 using Pearson correlations.

## 3 Results

Experiment 1 revealed a significant preference for AI-generated artworks. Without being provided with any information about the origin/authorship of the artworks, participants in the preference task indicated they preferred the AI-generated artworks significantly more often than the human-made artworks (AI-preference scores significantly above 50% chance-level) [*t*(126) = 5.39, *p* < 0.001, *d* = 0.48; Figure 2B].

Interestingly, when a separate group of participants in Experiment 2 were asked to detect which one of the two artworks was made by a computer, they could do so significantly better than chance. A one-sample t-test indicated AI detection accuracy was significantly above 50% [*t*(136) = 3.62, *p* < 0.001, *d* = 0.31] although a smaller effect size was observed compared to Experiment 1 (Figure 2B).

**Figure 3:**
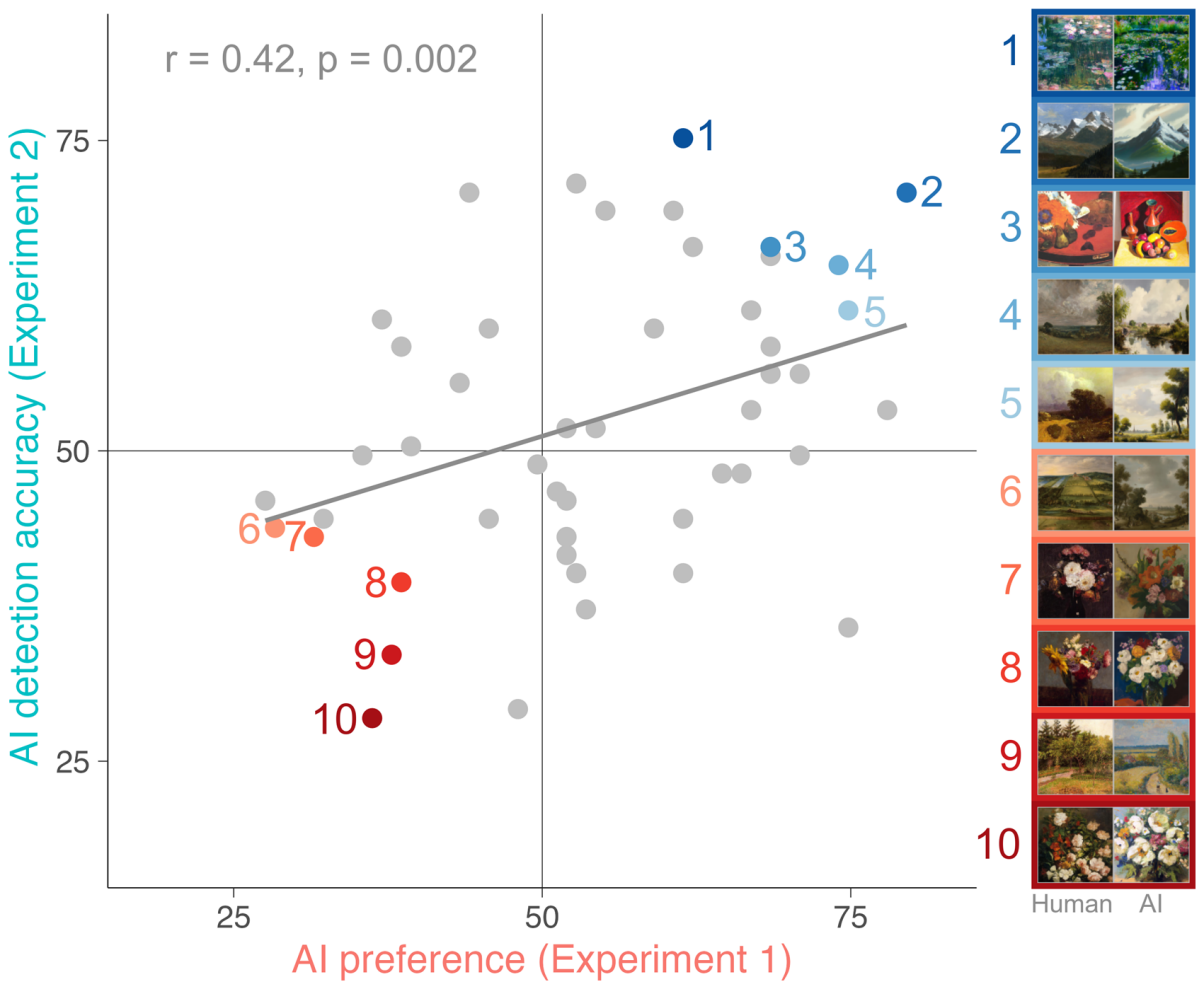
Correlation between AI preference scores in Experiment 1 and AI detection accuracy scores in Experiment 2. Each dot represents one human-AI artwork pair (50 in total). Coloured dots highlight the five pairs of artworks at both ends of the spectrum. The solid line represents the line of best fit.

Image-wise correlational analysis revealed a positive relationship between AI preference and AI detection accuracy scores associated with each pair [*t*(48) = 3.23, *p* = 0.002, *r* = 0.42]. As shown in Figure 3, the AI-generated artworks that Experiment 1 participants tended to prefer were also those that Experiment 2 participants were better able to detect, suggesting there may be features in the artworks driving both preference for and detection of AI-generated art. Importantly, this correlation remains almost identical when controlling for potential differences in mean luminance, root mean square contrast and entropy between human-made and AI-generated stimuli [*t*(48) = 3.15, *p* = 0.003, *r* = 0.42] suggesting that there are other more complex visual features influencing participants’ preference and discrimination.

Regarding the influence of expertise, our results indicated no significant correlations between participants’ experience in art (i.e., interest and knowledge in art, see methods for further details on how this was assessed) and AI preference and detection accuracy in either experiment [*r*(125) = 0.02, *p* = 0.79, and *r*(135) = 0.16, *p* = 0.06 for Experiments 1 and 2, respectively].

## 4 Discussion

There is a burgeoning sentiment that AI-image generation technology has reached a point of refinement that challenges our traditional understanding of the human perception and appreciation of art (Epstein et al., 2023; Oksanen et al., 2023). Our results evidence this claim, revealing that in the absence of attribution labels, human observers systematically prefer AI-generated artworks over stylistically similar artworks painted by real people. Should this effect prove universal, it could constitute a paradigm shift in art appreciation, favouring synthetic works over those created by human artists, which has the potential to transform the art world, while also raising new questions about authorship, authenticity, and the role of human creativity in the age of generative AI.

Our findings stand in contrast to prior research on subjective evaluations of computer-generated artwork, which have largely reported a negative bias towards AI art (Agudo et al., 2022; Caporusso et al., 2020; Chiarella et al., 2022; Gangadharbatla, 2022; Kirk et al., 2009). This work has primarily examined the role of authorship attribution in AI art perception, rather than the aesthetic value of the artworks themselves. Thus, the observed negative bias in these studies appears to relate to our explicit prejudice against artificially-generated content (i.e., if an artwork is labelled as computer-generated, we tend not to like it). In contrast, here we obtained observer preference decisions in the absence of any authorship label—a neutral presentation format that encouraged observers to judge the inherent aesthetic qualities of the artworks—and assessed authorship discrimination in a separate experiment. This approach allowed us to obtain a quantitative assessment of the degree to which observers prefer the inherent artistic qualities of real artworks made by humans vs. those created by new AI image-generation models, free from external biases.

Although observers in the first experiment consistently preferred artworks generated by DALL·E 2 over those made by human artists, it was not the case that these AI artworks were indistinguishable from human creations. In Experiment 2, a separate group of observers were asked to explicitly judge which of the two artworks in each pair was generated by a computer. We found they could reliably do so above chance-level. Moreover, there was a positive correlation between the image-pairs’ AI-preference and AI-detection scores, suggesting that the same visual features that made the AI-generated artworks more detectable to participants in Experiment 2 also made those artworks more appealing to participants in Experiment 1 (Van Geert and Wagemans, 2020). This intriguing pattern underscores the role that explicit bias against artificial creations has likely played in prior investigations (Agudo et al., 2022; Caporusso et al., 2020; Chiarella et al., 2022; Gangadharbatla, 2022; Kirk et al., 2009) of the aesthetic appeal of AI-generated artworks: When participants do not know the artworks are computer-generated, they freely prefer them. Interestingly, we found no evidence that these effects were moderated by observers’ art expertise, suggesting that the features in question are broadly accessible. However, our results suggest that they are not related to luminance, contrast and entropy, and that other visual features might have a more important role; a possibility which future research will no doubt explore in detail. This is an important research avenue together with examining the influence of contextual and authorship information on these effects to better understand the human perception of AI-generated artworks.

More generally, these results suggest that the technology behind DALL·E 2, in striving for stronger verisimilitude in computer-generated art, has evolved to do so by extrapolating (or exploiting) existing known biases in human cognition. On this thinking, DALL·E’s capacity to produce works that observers tend to prefer over human artworks could possibly be explained by the fact that its training dataset comprises images of artworks that are broadly popular and likely considered to be aesthetically pleasing. This is in line with recent research on “deepfakes,” wherein AI-generated faces not only fool observers with their hyper-realistic nature, but are also associated with enhanced perceptions of trustworthiness (Boháček and Farid, 2022; Groh et al., 2022; Nightingale and Farid, 2022). These findings raise critical concerns about the exact nature of the cognitive processes that could be targeted and manipulated using generative AI, and therefore, about its large-scale deployment without detailed investigation.

In a world increasingly shaped by the algorithms around us, the current findings suggest that as AI continues to evolve towards human capabilities, it may be poised to redefine our understanding of creative expression altogether. If AI-generated content can reach or even surpass aesthetic equivalence with human creation, the question of whether something can truly be considered “art” if it has no human architect becomes more complicated. Although AI image diffusion models currently still depend on some level of human intervention to produce artwork (from the initial production of training data to programming to user input), it seems increasingly likely that this necessity will decrease in future as the technologies progress. Our results are an initial step towards untangling the complex interaction between generative AI and human aesthetic preference; clearly, systematic examinations of AI-generated artworks’ features are needed to fully understand the mechanisms and implications of AI preferences. A clear future avenue for this field will be to combine parametric feature variations in AI-artwork with systematic manipulations of artwork-attribution (AI or human), to determine how low and mid-level visual featural differences interact with the documented negative bias associated with AI attribution (Chamberlain et al., 2018; Chiarella et al., 2022; Horton et al., 2023; Millet et al., 2023; Ragot et al., 2020). Indeed, this bias may well be shifting with the rapid adoption of AI technology in the wider population (Epstein et al., 2023; Oksanen et al., 2023; Prasad Agrawal, 2023).

In addition, it will be important to consider additional presentation parameters that may contribute to or modulate observer preferences: For example, here we presented artworks at a relatively small size, cropped from their original dimensions to conform to a 1:1 ratio. Since these aspects necessarily change the composition (and therefore appreciation) of the artwork, it will be necessary to explore whether our results hold under different presentation formats, as well as across real-world and digital presentation formats. Digital presentation might have favoured AI-made works. Some critical features in human-made works such as texture might have been lost with their digital representation. Furthermore, while specific efforts have been made to account for selection bias using image statistics, our design required some degree of curation to omit clear outliers produced by DALL-E 2 that did not match the criteria laid out in advance. Critically, this means that our results do not automatically apply to the state of AI-image generation as a whole, but rather illustrate the height of its capabilities. In juxtaposing samples of the acme of traditional art with that which AI is capable of producing, we get a better view of the perception of AI as it continues to progress. Future studies should expand on this further by including a broader selection of images and removing the potential for selection bias through human intervention entirely. Importantly, excluding famous human-made artworks might have favoured AI artworks. Including famous human-made artworks assuming they could have been tested on completely naïve participants with no previous exposure could have increased preference for human-made works. There might be features in these artworks resulting in higher preference independently of how famous they are.

Moreover, future research could investigate the effects of expertise further as this was only succinctly assessed in this online experiment across three generic questions. Variations in the type of expertise and the number of years could be more deeply examined. Demographics such as age and cultural background could also be explored. They might influence how participants perceive and appreciate these artworks and the features they might focus on. It can also be noted that the AI-preference we report here is, by design, derived from a constrained set of artistic styles. As such, future work expanding this line of investigation could look to systematic comparisons of different styles. These are necessary steps for future research to reach a broader understanding of the perception of digital artworks in the general population. This has implications for the art market, museums, curators, and future artists, with new insights into the type of artworks, in the digital space in particular, people might prefer. It might also be important to extend this research in future studies to other creative domains. The production of movies and music, for instance, are all changing quickly with generative AI. Understanding similarities and differences across domains, could help identify universal mechanisms in this new generation of AI models biassing human perception and preference.

To conclude, our findings suggest that we might be observing with recent advances in AI technology a shift in art preference to favour synthetic creations, raising critical questions about the way we think about art and its value to our society. As the field of generative-AI continues to accelerate—spurring equal parts concern and excitement—there can be no doubt as to the urgency in this challenge. DALL·E 2 will soon be superseded by the next generation of algorithms with as-yet unknown capabilities. Understanding how the human experience intersects with this technology will be critical to ensuring its positive impact on our society.

## Data Availability

The datasets presented in this study can be found in online repositories. The names of the repository/repositories and accession number(s) can be found at: https://osf.io/n7w32/.

## Footnotes

^1^ https://openai.com/dall-e-2

^2^ https://commons.wikimedia.org

^3^ https://www.wikiart.org

^4^ https://openai.com/dall-e-2

^5^ https://commons.wikimedia.org

^6^ https://www.wikiart.org
