# Supplementary material for: Human perception of art in the age of artificial intelligence
Source: Front Psychol. 2025 Jan 8;15:1497469. doi: 10.3389/fpsyg.2024.1497469 (PMC11750838; doi:10.3389/fpsyg.2024.1497469)
Supplement: Supplementary file 1 [file Data_Sheet_1.docx]

Supplementary Material


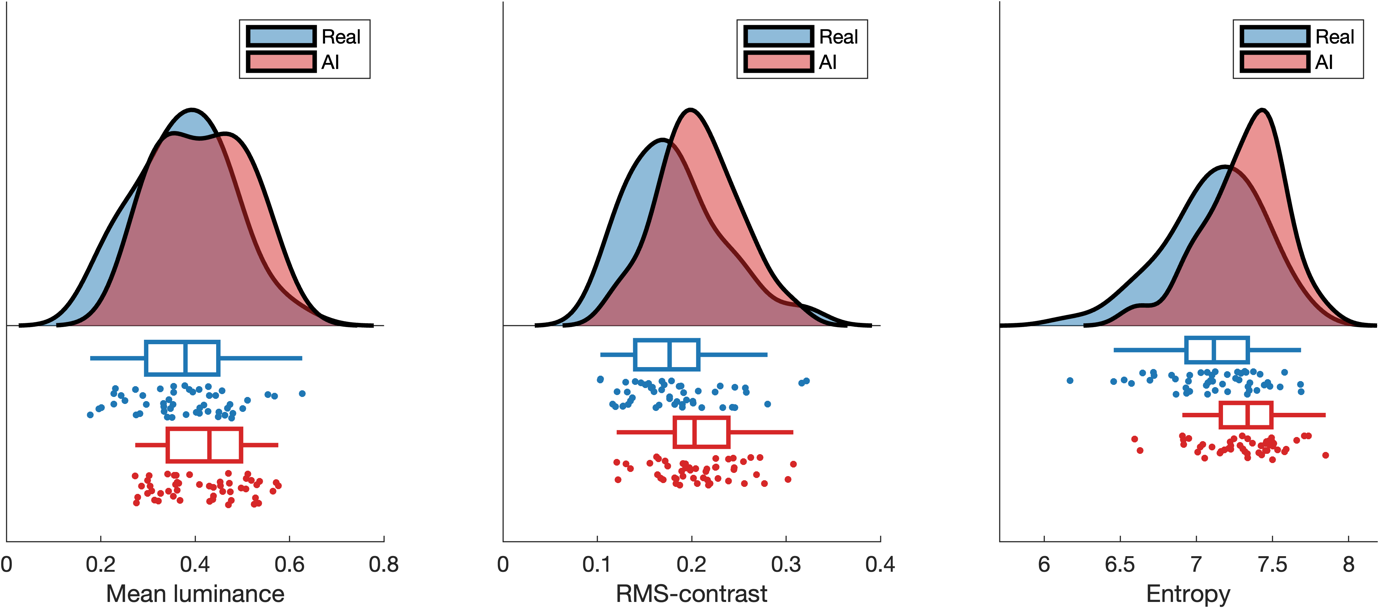


**Figure 1**. Mean luminance, root mean square contrast and entropy for human-made and AI-generated artworks. Distributions, boxplots and individual image values are represented in blue and red for the two categories of artworks.
